# A spatiotemporal analysis of the food dissemination process and the trophallactic network in the ant *Lasius niger*

**DOI:** 10.1038/s41598-019-52019-6

**Published:** 2019-10-30

**Authors:** Joffrey Planckaert, Stamatios C. Nicolis, Jean-Louis Deneubourg, Cédric Sueur, Olivier Bles

**Affiliations:** 1 Center for Nonlinear Phenomena and Complex Systems (Cenoli) - CP 231, Université libre de Bruxelles (ULB), Campus Plaine, Boulevard du Triomphe, Building NO - level 5, B-1050 Bruxelles, Belgium; 2 Université de Strasbourg, CNRS, IPHC, UMR 7178 Strasbourg, France

**Keywords:** Animal behaviour, Entomology

## Abstract

Intranidal food dissemination through trophallactic exchanges is a fundamental issue in social insect colonies but its underlying mechanisms are far from being clear. In light of the division of work, network theory and collective food management we develop a framework to investigate the spatiotemporal dynamics of the trophallactic network in starved *Lasius niger* ant colonies. Thanks to tracking methods we are able to record spatial locations of the trophallactic interactions in the nest. We highlight quantitative differences between the foragers and non-foragers concerning their contributions, their roles (donor/recipient) and their spatial distributions. Moreover, at the intracaste level, we show interindividual differences in all activities and we characterise their nature. In particular, within each caste, all the individuals have the same probability to start their food exchange activity but their probability to exchange differs after their first trophallactic event. Interestingly, despite the highlighted interindividual differences, the trophallactic network does not differ from a random network.

## Introduction

Eusociality is one of the most complex and integrated forms of social life, implying coordination in broodcare behaviour, overlapping generations and polytheism (i.e., the existence of fertile and non-fertile castes). Moreover, division of work also occurs within the non-fertile caste^1^ and relies on different factors (e.g., age, morphology, genetics or individual experiences) and on their interplay (e.g.^2^). Eusociality implies colony-level coordination through information sharing and exchanges of material and food at the individual level. Food retrieving and food dissemination through the colony implies division of work between a small fraction of the colony (the foragers) that leaves the nest to explore the environment, harvests food and brings it back to the nest while the rest of the colony (the non-foragers) stays inside the nest. Upon returning to the nest after having retrieved food, the foragers share their crop contents through trophallactic interactions (mouth-to-mouth exchanges) with nestmates that are in turn able to share the food. These food exchanges are modulated by colony needs and the level of satiation^3,4^.

Trophallactic events are not only a way of delivering food to the colony members that do not feed at the food source^1^ but are also a way of diffusing information (such as nutritional needs at the individual and colony levels) and biological material (such as symbionts and hormones)^5^. Starved ants inside the nest beg for food from their neighbours, who in turn, are able or not to satisfy these requests. These behaviours result in “chains of demand” that lead to the emergence of the trophallactic cascade from laden individuals to starved individuals, according to a continuum of load^6–8^. Such a pattern of food dissemination involving consecutive transfers of material from one individual to another is assumed to be more efficient and results in a more homogeneous distribution than direct transfers from only the original donors^9^.

Moreover, divergences could emerge concerning the information held by the different castes: while the foragers hold information about the food availability, the non-foragers hold information about the colony needs and food store level, e.g.^6,10,11^. Therefore, numerous exchanges and communications occur between forager and non-forager individuals, particularly at the interface between the nest and the environment^12,13^, which regulates the food flow that enters the nest to meet the colony needs. In ants, these regulations are the by-product of the interaction between gradually satiated non-foragers and foragers. Increasing the crop contents of non-foragers slows the rate of food transfers from foragers to non-foragers, which in turn modulates the decision of the forager to leave the nest^3^. This simple mechanism of food flow regulation cannot be generalised to other social insects such as honeybee in which more complex mechanisms of regulation are involved^14–16^.

In addition, the unloading behaviour of the donor is also modulated by the colony state. In starved colonies, a single donor transfers almost all its crop content to approximately 100 workers, while in fed colonies, the donor only transfers less than a third of its crop content, but its food reaches approximately the same number of ants^17^. Furthermore, colony size, division of work and spatial occupation patterns as well as interactions among these factors affect food sharing behaviours and the dynamics of information flow^9,18–21^. Thus, food collection and distribution behaviours do not simply result from hunger responses at the individual level but rather result from a complex interplay between the nutritional needs at the colony level and the decisions of individuals.

Despite the central role of the trophallactic interactions in the regulation of food flow in social insects, the way the chains of demand and the spatial organisation of food transfers within the colony are established are still largely ignored^22–25^. Most of the works on this subject have focused on the food flow dissemination inside the nest^26,27^ and the quantity received by different castes^28^ but have ignored the individuality and identity of the trophallactic partners^7^. The role and modulation of individual trophallactic activities have only been recently investigated in ants^3,29,30^.

In this context, we combined methodological and analytical tools allowing for quantitative analyses that complement and unify previous studies carried out collective food management in several ant species^3,30–32^. Our main objective was to characterise the food dissemination process by establishing how its spatiotemporal dynamics, the division of work (at the individual and caste-level) and the subsequent network of food exchanges in ant colonies are intertwined. To this end individuals were classified into castes based on whether they visited the food source (foragers) or did not (non-foragers) and we recorded the spatial position and the identity of the donor and the receiver of the oral food exchanges inside the nest. Several metrics as well as simulations were utilised to quantify the (intra- and inter-caste level) heterogeneity of the involvement to the trophallactic activity and the resulting trophallactic networks. Finally, different analyses were performed to identify the origin of this heterogeneity.

## Methods and Materials

### Ant colony set-up

From five large mother colonies (>1000 ants) of *Lasius niger* (collected in Brussels, Belgium, autumn 2016), we created five queenless and broodless subcolonies of at least 50 randomly chosen workers. The ants were individually labelled with ArucoColor tags (https://sites.google.com/site/usetrackerac/), allowing automatic identification of the ants. Each tag was stuck to the abdomen and had a side length of 0.8 mm, weighed 0.1 mg (corresponding to less than 5% of the average mass of an adult worker or less than 10% of the amount of food a worker carries^33^) and was printed on waterproof paper at a resolution of 1200 dpi. The tags were hand-cut using a scalpel and a steel ruler as guide. Following a 5-min acclimatisation period, the labelling was not observed to impede the ants’ behaviours, movements or interactions. Each subcolony was introduced to the experimental set-up between 15 to 18 days prior to the first experiment; each set-up was composed of a one-chamber nest (56 × 41 × 2 mm) covered by a glass window. This acclimation period was long enough to stabilise the task repartition between individuals. A single access portal (4 × 3 × 2 mm) lead to the foraging area (61 × 49 × mm) containing a 0.3 M sucrose solution and water *ad libitum* (Fig. S1). The walls of the foraging area were covered in Fluon® to prevent the ants from escaping. The subcolonies were kept at 22 ± 3 °C and 60 ± 5% relative humidity, with a 12:12 h constant photoperiod.

### Data collection

After 4 days of starvation, we introduced 3 mL of 1 M sucrose solution. The ants were filmed for 90 mins, starting 30 mins before the food source introduction. Each colony was tested once. The video data were recorded using a Panasonic® Lumix DMC-GH4-R mounted with a 30 mm Olympus® ED lens capturing 25 frames/s at the definition of 4180*2160 p. We discriminated foragers (Fs) from non-foragers (NFs). An individual was considered as a forager if it spent at least 5 consecutive seconds feeding at the food source during the experiment. Additionally, at each minute, we conducted a scan-sampling^34^ of all the trophallactic interactions inside the nest, identifying the donor, the receiver and the X and Y spatial positions of the trophallactic events (contact point of the mandibles of both ants). A trophallactic event was recorded when ants engaged in mandible-to-mandible contact for greater than 5 s. Even if the food flow was not directly observable previous studies^26,31^ showed that the directionality of the food flow and the role of the donor and the receiver are determined by the characteristic body posture and the mandibles positions (see also Fig. S2). A trophallactic event involving the same individuals on two or several consecutive scans was considered as a single trophallactic event of 2 or several min lengths. As a strong correlation was observed between the total time spent during trophallactic events and the number of trophallactic events (Fig. S3, Spearman, R² = 0.69, *p* < 0.01 in each case), we focused on the number of trophallactic events (a proxy for the amount of food transferred) for all the analyses.

### Statistical and social network analysis

**Figure 1:**
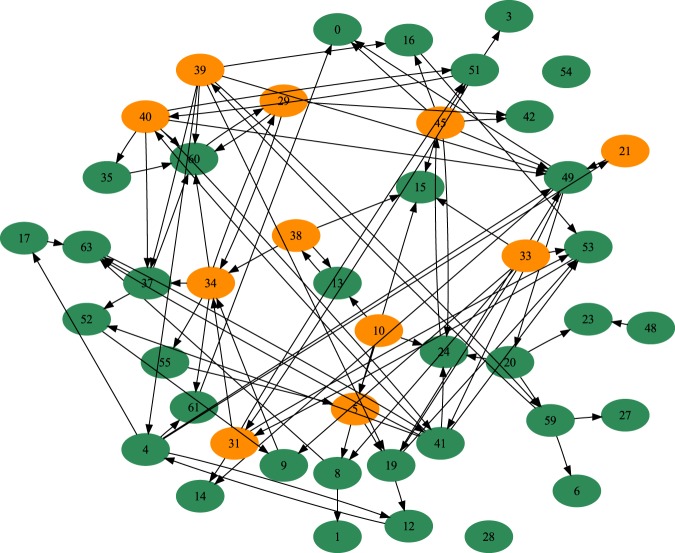
Example of an aggregated trophallactic network. Node = individual, directed black arrow = trophallactic exchange from the donor to the receiver. Orange = foragers, green = non-foragers.

We checked the homogeneity of the mean number of trophallactic events in the colonies (as well as the mean number of trophallactic events where food was given and received by foragers and non-foragers) by carrying out a two-sided Kruskal-Wallis one-way analysis of variance (hereafter “KW”). The Mann-Whitney (hereafter “MW”) test was used to compare the mean number of trophallactic events (given/receive) between foragers and non-foragers. The experimental distributions of the number of trophallactic events between castes were compared using a Kolmogorov-Smirnov (hereafter “KS”) test. The complete trophallactic network of each subcolony was built (e.g., Fig. 1). In this representation, each node corresponds to individuals, and an edge represents trophallactic events directed from the donor to the receiver.

We performed weighted and directed analyses. Our network analyses were performed at both the individual level and functional category (forager/non-forager) level. We calculated the degree centrality, eigenvector centrality, betweenness centrality and closeness centrality of each individual. Degree centrality is based on individuals’ number connections and can be seen as a general measure of how social an individual is^35^. The betweenness is an estimator of how important an individual ant is for promoting connectivity across the entire colony and is measured by the number of times an individual acts as a bridge along the shortest path between two other ants^36^. The closeness is based on the shortest paths from an individual to every other individual; the more central an ant is, the lower its total distance is from all other ants^35^. The eigenvector is a value accounting for the centrality of a node’s neighbours^37^. The clustering coefficient determines the existence of “communities” in a network, such as node pairs with many more edges between them than other node pairs^38^.

At the global level, the efficiency of the trophallactic network, defined as the multiplicative inverse of the shortest path distance between all pairs of nodes, was calculated. The heterogeneity of the distribution of the trophallactic activity among all the workers of each experiment (N = 5) was quantified using the Lorenz curve and the Gini coefficient. Such a curve displays the share of trophallactic activity (Y axis) accounted for by the top x% of workers (sorted by the number of trophallactic events performed per individual) in the colony. A perfectly equitable distribution of foraging activity would correspond to the line Y = X. The Gini coefficient is known as the ratio between the area below the experimental Lorenz curve and the triangular area below the perfect equality case Y = X and provides a measure of the degree of inequality in the distribution of trophallactic activity, ranging from 0 (perfect equality) to 1 (perfect inequality).

To estimate whether the observed Gini coefficient values and the social network metrics experimentally measured were different from those under random expectations, each empirical network was compared against an ensemble of N = 1000 randomised networks created by randomly rewiring all the edges between all the nodes, destroying all the features of the original network (Full Random network = FR)^39^. The experimental proportion of each type of trophallactic couple (F- > F, F- > NF, NF- > F and NF- > NF) was compared with the ones generated by the FR (these were only based on the relative proportion of the Fs and NFs workers).

We also tested the relation between the number of trophallactic events to the time of the first trophallactic event. Assuming that all individuals are identical, the distribution of the number of trophallactic events by individuals starting at the same time is a Poisson distribution. This theoretical distribution was compared to the experimentally observed one using a dispersion test (Poisson GLM). Similar test was applied to characterise the distribution of the number of visits of foragers at the food source.

To evaluate whether the temporal structure of the trophallactic network facilitated spreading, the empirical network was compared against an ensemble of N = 1000 temporally randomised networks. This ensemble was created by randomising the original trophallactic events with a randomly permuted times (RP) reference model^39^, which shuffled the times among the original trophallactic contacts. Temporal randomisation destroys temporal correlation but maintains all other features, including the number of trophallactic events and nodes and the topology of the original network leading to the creation of a temporally randomised network. To assess the effect of network temporal structure on the spreading speed, we compared the time when 50% of the ants performed there first trophallactic events in the empirical network and the theoretical corresponding one.

To evaluate the spatial distribution of the trophallactic events at the individual level, we calculated the spatial position of the gravity centre of the polygon resulting from all the trophallactic events for each individual. Then, still at the individual level, we measured the average distance of each trophallactic event from this gravity centre and compared it between all the foragers and non-foragers.

A Z-test (hereafter “ZT”) was then performed to evaluate the significance of the differences between the observed and random metrics from the FR and RP reference networks. To estimate the density and the spatial location of ant aggregates inside the nest, we divided the nest area into 30 cells of equal area (9.33*8.22 mm, equivalent to two ants length) in which the number of ants was automatically counted and accumulated by the USETracker software every 10 mins over the duration of the experiment. All analyses were conducted with Python 3.6 with the NetworkX.2.1, PyGraphviz 1.4, NumPy 1.14, SciPy 1.0.0 and Matplotlib 2.2 packages. The threshold for significance was set at *p* < 0.05.

## Results

### Global results

The subcolonies (N = 5) were composed of 53.4 ± 5.2 ants of which 44.2 ± 1.6 ants were active trophallactic participants (participated in at least one trophallactic event) and performed a total of 99.0 ± 17.4 trophallactic interactions. A summary of the trophallactic activity at the colony and caste levels is given in Table 1 (see also Table S1). The colonies were homogeneous in terms of trophallactic activity/network parameters and caste composition. Therefore, for clarity, we merged and averaged the experimental results of the 5 colonies in the rest of the paper (Table S2).

**Table 1:** Details of the trophallactic activity and social network metrics per colony and caste. Values = mean from experiments (N = 5), parentheses = s.d. See Table S1 for more details.

		Foragers	Non-foragers	Mann-Whitney
	Number of individuals	12.2 (1.9)	41.2 (5.9)	*p* = 0.006 (*U* = 0.0)
Number of trophallactic events	Total per colony	77.4 (22.7)	120.6 (16.5)	*p* = 0.001 (*U* = 8.0)
	as donors	60.2 (15.2)	38.8 (8.9)	*p* = 0.02 (*U* = 2.5)
	as recipients	17.2 (8.0)	81.8 (9.7)	*p* = 0.006 (*U* = 0.0)
	Mean per colony	6.3 (2.9)	2.9 (2.7)	*p* < 1.10-5 (*U* = 0.0)
	as donors	4.9 (2.7)	0.9 (1.4)	*p* < 1.10-5 (*U* = 1194.0)
	as recipients	1.4 (1.4)	2.0 (1.8)	*p* = *0.012* (*U* = 5113.0)
Social network metrics	Betweenness	0.06 (0.06)	0.03 (0.04)	*p* < 1.10-5 (*U* = 2319.0)
	Closeness	0.33 (0.23)	0.3 (0.34)	*p* = 0.001 (*U* = 3049.0)
	Eigenvector	0.15 (0.09)	0.11 (0.07)	*p* < 2.10-5 (*U* = 2324.0)
	Clustering	0.11 (0.15)	0.09(0.20)	*p* = 0.001 (*U* = 3653.5)

### Individual trophallactic activity and global pattern of exchanges between foragers and non-foragers

**Figure 2:**
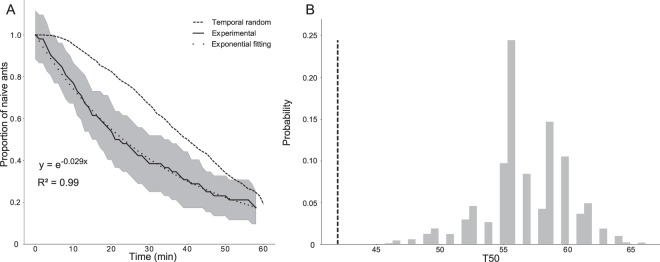
(**A**) Survival curves of the proportion of the naïve ants (that still had not performed any trophallactic events) in the experimental networks (full black line = mean from experiments, grey area = standard deviation, N = 5 experiments) and in the RP reference networks (temporal randomisation, dashed black line, mean from N = 1000 for each experiment). Dotted black line = exponential fitting. (**B**) Example from one experiment: empirical (vertical dotted line) and theoretical distributions (grey bar, from 1000 RP reference networks) of the T_50_ for the half of the population that performed at least one trophallactic exchange, ZT: Z = −4.51, p < 0.0001. See Fig. S4 for details of the other experiments.

**Figure 3:**
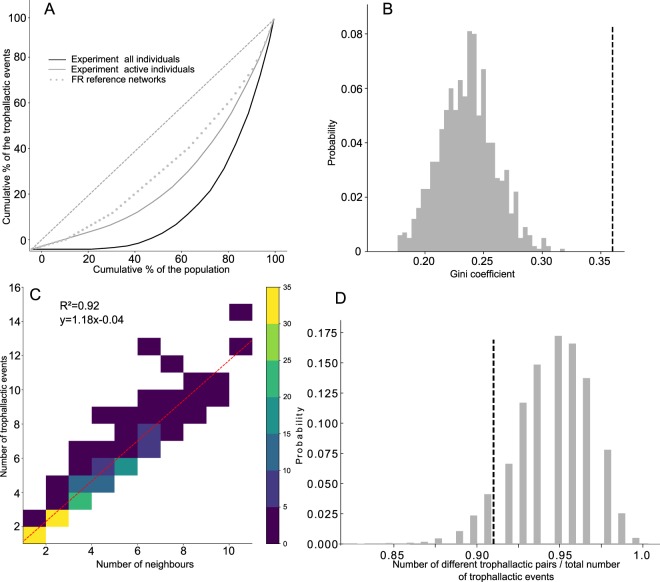
(**A**) Mean Lorenz curves showing the cumulative percentage of all trophallactic events (y axis) vs. the percentage of the population (x axis, sorted by the number of trophallactic events performed per trial per each worker), when considering all the colony members (active and inactive, black line) or only the individuals having performed at least one trophallactic event (active, grey line). The grey dotted line represents the distribution activity from the FR network. (**B**) Distribution of the Gini coefficient measured in N = 1000 FR reference networks (grey bars) and the experimentally measured value of all active ants (vertical dashed line) in one experiment. See also Fig. S6. (**C**) Correlation between the number of trophallactic events and the number of neighbours, for each individual. The colour bar indicates the number of individuals. (**D**) Ratio between the total number of trophallactic pairs and the total number of trophallactic interactions. Vertical dashed line = mean experimental ratio (N = 5). Grey bars = theoretical distribution from FR reference networks (N = 1000).

The population of ants that had not yet been involved in a trophallactic event (naïve ants) decreased exponentially as a function of time with a mean time equal to 35 mins (Fig. 2A, Spearman, R² = 0.996, p < 0.001). This suggests that the individual probability of joining the trophallactic network was constant and homogeneous through the colony. This decrease was faster in the experimental network than in the RP networks, which were temporally randomised (Fig. 2B, ZT, p < 0.001 in each case, see also Fig. S4). In each experiment, the Lorenz curves showed a strong heterogeneity in the distribution of the trophallactic activity within the ants: ~20% of the total population performed more than 60% of the trophallactic events (Fig. 3A). This heterogeneity was more marked than the theoretical heterogeneity obtained from purely random exchanges, such as in the FR networks, even when we discarded the data from the inactive ants (Fig. 3A). In every experiment, the Gini coefficient was significantly larger than the one resulting from purely random exchanges (Fig. 3B, ZT, *p* < 0.04 in each case, see also Fig. S5). There was a strong heterogeneity in the number of partners of trophallactic events and a linear correlation between the number of trophallactic partners and the number of trophallactic events performed (Fig. 3C, R² = 0.92, *p* < 0.05). Most of the individuals performed only one or two trophallactic events during an experiment (Fig. 3C). The number of times each trophallactic pair met did not differ from a FR network distribution (Fig. 3D, ZT, *p* = 0.38).

### Intracaste distributions and characteristics of the trophallactic activity

**Figure 4:**
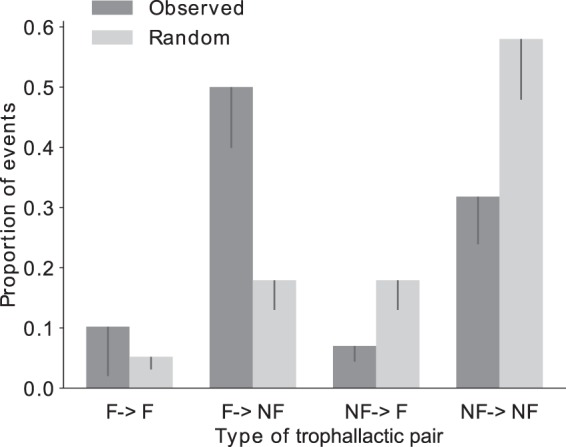
Comparison of the theoretical distribution of the trophallactic events based on a homogenous repartition of the trophallactic events between all the ants with observed distributions.

**Figure 5:**
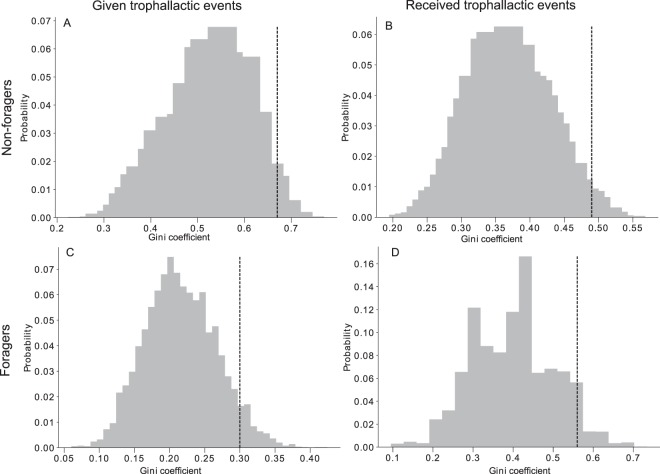
Mean observed (vertical dotted line, N = 5) and theoretical distribution (grey bar, N = 5 * 1000) of the Gini coefficient of the trophallactic events given (Fig. 5A, ZT, Z = 1.91, p = 0.028) and received (Fig. 5B, ZT, Z = 2.00, p = 0.022) by the non-foragers and the trophallactic events given (Fig. 5C, ZT, Z = 1.49, p = 0.067) and received (Fig. 5D, ZT, Z = 1.45, p = 0.073) by the foragers.

The experimental proportions of given and received trophallactic exchanges between both foragers and non-foragers were different from the theoretical proportions resulting from the FR networks, taking into account the proportions of foragers and non-foragers (Fig. 4. χ²; *p* < 1.10–4). The experimental Gini coefficients of both given (Fig. 5A) and received trophallactic events (Fig. 5B) among the non-foragers were significantly (ZT, *p* = 0.028 and *p* = 0.022, respectively) different from the distribution of the Gini coefficient in the FR networks where all the non-foragers are identical and have the same probability to exchange food. Following the same procedure for the foragers, the Gini coefficient tended to be different (ZT, *p* = 0.067 and *p* = 0.073, respectively; see Fig. 5C,D for details). Moreover, the numbers of given and received trophallactic events are correlated in the case of non-foragers while they are not in the case of the foragers (Fig. S6A,B, respectively p = 1.10–3 and p = 0.238).

**Figure 6:**
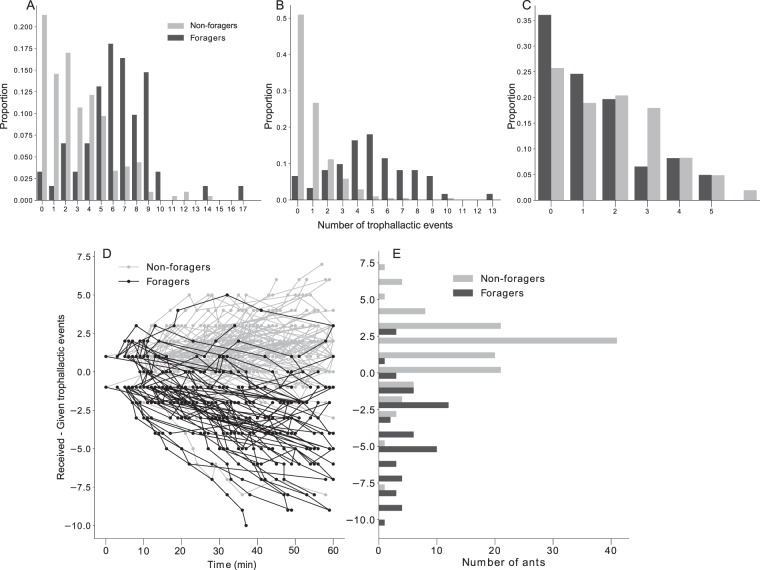
Distribution of the number of trophallactic events done within non-foragers (NF) and foragers (F). (**A**) All the trophallactic events. (**B**) Given trophallactic events. (**C**) Received trophallactic events. (**D**) Dynamical trophallactic events statement of each individual. (**E**) Distribution of the final statement of non-foragers and foragers, KS, D = 0.76, p < 1.10-5.

A larger portion of the non-foragers did not participate in any trophallactic events compared to the foragers, and the distributions of the total number of trophallactic events of foragers and non-foragers were different (Fig. 6A, KS, *p* < 0.0001). The distributions of the given (received) trophallactic events of foragers and non-foragers were different (similar) (Fig. 6B, KS, *p* < 0.0001; Fig. 6C, KS, *p* = 0.1632). The number of trophallactic events given was linearly correlated to the number of visits to the food source; on average, after a visit at the food source, a forager participated in two trophallactic events (Fig. S7A), and the later a forager visited the food source for the first time the fewer times she would subsequently visit it (Fig. S7B). The dispersion test (GLM Poisson) did not reveal an overdispersion in the number of visits at the food source (p = 0.34). Additionally, the later a forager/non-forager given/received her first trophallactic event, the fewer times she would give/receive food through a trophallactic interaction (Fig. S7C–F). This relation is linear and the slope is the mean rate of trophallactic activity. The rates of receiving and giving are not different for the non-foragers (p = 0.37) while the giving rate of the foragers is three times greater than the receiving rate (p = 0.007). Also, the dispersion test revealed an overdispersion in the experimentally observed trophallactic activity respectively for the foragers/non-foragers and for their given/received trophallactic interactions (GLM Poisson, p < 0.001 in each case). The analysis of the balance of the trophallactic events (given minus received) of each individual revealed that the foragers gave food through more trophallactic events than they received while the non-foragers received more food than they gave (Fig. 6D,E, KS, *p* < 1.10–5).

To summarize, these results highlight inter-individual differences for each activity within each caste. At the individual level, these differences between given and received trophallactic events are correlated in the case of the non-foragers (Fig. S6A) while the given trophallactic events are correlated to the number of visits to the food source in the case of the foragers.

### Social network analysis and dynamics of food dissemination

The foragers had higher betweenness, closeness, eigenvector and clustering coefficients than non-foragers (Table 1, MW, *p* < 0.002 in each case). At the colony level, none of these parameters nor the efficiency parameter differed from the ones measured in the randomised FR networks (Figs S8–12, ZT, *p* > 0.5 in each case). The T_50_ of the first given trophallactic events of foragers (41.6 ± 1.8 min) was significantly lower than the T_50_ of the first given trophallactic events of the non-foragers (66.0 ± 6.7 min, MW, *p* = 0.0058, Fig. S13A). This difference is still relevant for the T_75_ (MW; *p* = 0.0059) and T_95_ (MW, *p* = 0.01). The T_50_ of the first received trophallactic events of the foragers (45.0 ± 1.7 min) was also significantly lower than the T_50_ of the non-foragers (57.0 ± 3.7 min, MW, *p* = 0.0056, Fig. S13B). This difference was not relevant when considering the T_75_ and T_95_ (MW, *p* > 0.264 in each case).

### Analysis of the spatial distribution of the trophallactic events

**Figure 7:**
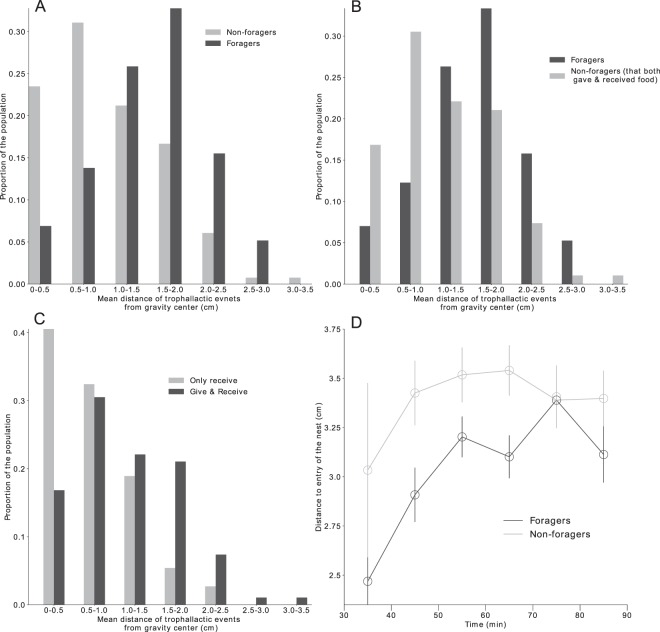
(**A**) Distribution of the mean distance of each trophallactic event from the gravity center of all the trophallactic interactions per ant. (**B**) Distribution of the mean distance of trophallactic events from the gravity center of non-foragers considering whether they only received or gave and received food during trophallactic events. (**C**) Distribution of the mean distance of trophallactic events from the gravity center for foragers and non-foragers who gave food in at least one trophallactic event. (**D**) Distribution of the distances of the trophallactic events initiated relative to the entrance of the nest over time.

Trophallactic events inside the nest were non-homogeneously spatially distributed (Fig. S14, ZT, *p* < 0.0002). The spatial locations of the trophallactic events and aggregates of ants were significantly correlated (Fig. S15). The foragers gave 33.2% (100/301) of their trophallactic events in the foreground part of the nest (the half the nest next to the nest entrance), a percentage significantly higher than that of the non-foragers (17.8%, 42/236, χ², *p* = 0.0074). The mean distance of the trophallactic events performed by an ant to the gravity centre of the polygon resulting from the spatial position of all its trophallactic interactions (see material and method section) of the foragers (1.49 ± 0.58 cm) was significantly larger than that of all the non-foragers (1.01 ± 0.65 cm) (Fig. 7A, MW, *p* = 1.2 10^−6^) and that of the non-foragers that gave and received food (MW, *p* = 0.00021; Fig. 7B). This last mean distance (1.13 ± 0.66 cm) was significantly larger than the mean distance of the non-foragers that only received food (Fig. 7C, only receive = 0.71 ± 0.53 cm, MW, *p* = 7.4 10^−4^). Between the first 10 mins and the last 10 mins of the experiments, the distance to the nest entrance where trophallactic events occurred increased (from 2,75 cm to 3,25 cm for the foragers; from 3 cm to 3.5 cm for the non-foragers) (Fig. 7D).

## Discussion

In this study, we quantitatively analysed the dynamics and the network of food dissemination in colonies of *L. niger* provided with a food source (1 M sucrose solution) after 4 days of starvation. The experimental food spreading rate inside the nest, as reflected here by the involvement rate of individuals in trophallactic activity is approximately 50% faster than in a temporally randomised network. Note that this result has also been shown in honeybee colonies^40^. We developed a methodological and analytical framework of food dissemination processes in ant colonies. It complements and somehow improves previous frameworks in terms of individual tracking^30^, and in terms of network analysis^3,32^. Indeed, we automatized individual tracking through the 90 minutes of experiment and collected not only spatial variables like trophallactic event positions, but also assessed the roles of each individual in the food dissemination process and view it as a social network whose properties can be quantified.

At the colony level, the observed heterogeneity of the trophallactic activity among all individuals is in agreement with most of the literature on the activity distribution among eusocial insect workers^32,41–44^. Additionally, at the individual level, no link was established between the number of given and received trophallactic events. The linear relationship between the number of trophallactic partners and the number of trophallactic events per individual suggests that there are no privileged pairs, most of them being observed once. Moreover, the comparison between experimental pairs and the ones resulting from theoretical simulations revealed that these pairs are randomly created. This last result shows that the rules governing the pair formations are not at the origin of the difference between the food spreading rate in the observed and randomised networks. Therefore, the individuals inside the nest seem to be anonymous for their conspecifics^45^ and interact opportunistically without apparent individual recognition. The well-known spatial fidelity in ants^20^ could have led to privileged trophallactic partners, even in the absence of individual recognition, a phenomenon that did not occur in our experiments. These random encounters are likely to contribute to the resilience of the trophallactic network^40^.

Several differences were observed between the trophallactic activity of the castes of foragers and non-foragers, although this classification only results from the rough criterion according to which an individual feeding to the sucrose source at least once is considered as a forager. A noticeable difference concerned the individual rate of donation which was larger for foragers. As for the food acquisition rate, it was quite similar between the castes. At the intracaste level, the computation of the Gini coefficients showed that the distribution of the number of given and received trophallactic events performed by the non-foragers (foragers) is (tends to be) more heterogenous than the ones resulting from the randomised networks. The exponential distribution of the 1^st^ visit at the food source and the linear correlation between the numbers of visits (which are Poisson distributed) and the times of the first visits to the food source showed a homogeneous participation of the foragers to food collection. The exponential distribution of the times of the first trophallactic event suggests that all individuals from both castes start with the same constant probability. A dispersion test revealed interindividual differences at the level of the total number of given and received trophallactic events in both castes. All in all, these results strongly suggest that the individuals do not differ in the probability to start food exchange but do differ once they are engaged in food distribution. The correlation between given and received trophallactic events only found in non-foragers may be the result of the fact that these individuals do not only store food but also redistribute it. The existence of a gradient of trophallactic interactions could mean that a gradient of division of labour is at work, where individuals which are lowly engaged in food exchanges may still be available to complete other tasks.

The trophallactic network and the global dynamics of the food exchanges were the result of the interactions between castes and idiosyncratic individuals. In this network, the foragers had a higher trophallactic activity (number of trophallactic events per individual), exchanged food faster and had larger network indexes (betweenness, closeness and eigenvector coefficients) than the non-foragers. This leads to the conclusion that the foragers not only brought the food into the nest but also occupied a central position in the network. In other words, foragers were the major actors in food dissemination within the nest. Foragers represented 20% of the population, while they performed more than 60% of interactions as donors. Approximately 50% of the trophallactic interactions occurred between foragers and non-foragers, but only 30% of the food exchanges occurred within the non-foragers. Despite the major role of the foragers and the heterogeneity in the trophallactic activity, the high level of randomness in the trophallactic pair formation may prevent highlighting any differences between the empirical network indexes and those characterising random networks. In addition to the social position of individuals within the trophallactic network, we were also interested in how trophallactic interactions are spatially distributed. A correlation between the spatial location of the trophallactic events and ant aggregates was also highlighted, suggesting a dependence between the spatial patterns of the ants and the trophallactic network. This correlation could result from the interplay between two different spatial behaviours specific to the foragers and non-foragers, the non-foragers tending to be gregarious and the foragers having a more exploratory behaviour. The foragers’ mean distance of the trophallactic events to their gravity centre was longer than the one of the non-foragers that gave food at least once, which is longer than the one of the non-foragers that only received food. This is an agreement with the fact that the foragers that brought food back to the nest acted as donors more frequently at locations close to the nest entrance than did the non-foragers. After 30 mins of food collection, both foragers and non-foragers performed their trophallactic interactions in the same area, far from the nest entrance. This is in agreement with previous studies showing a similar evolution of the spatial distribution of trophallactic interactions along with colony satiation^3,30^.

Through the quantification of trophallactic activity and the resulting network of food exchanges inside the nest, we have gained insights on how food retrieval is organised at the individual, caste and colony levels. The “rough” classification of foragers / non-foragers, based on at least one visit to the food source, proved to be relevant because it highlighted differences in the food-exchange behaviours between these two groups inside the nest. Similarly, the time of the first visit to the food source or the time of the first trophallactic interaction turned out to be a determining factor at the origin of the heterogeneity of the trophallactic activity. At the spatial level, we showed that the foragers go deeper from the entrance of the nest as a function of the colony satiation, which is in agreement with previous studies^30,31^. We provided also complementary results uncovering different occupancy patterns between the non-foragers and the foragers, the latter displaying larger food area distributions. Furthermore, the trophallactic exchanges were correlated with the spatial positions of the ant aggregates, potentially facilitating trophallactic pair formations^4^. Moreover, at the colony level, we found no difference between the empirical and randomised trophallactic networks, indicating the absence of marked social structure. The random character of the empirical network could be at the basis of a robust food dissemination process. However, it is important to note that our results could be due to our experimental setup which was characterised by a one-chamber nest and one 1 M sucrose food source with colonies composed of a small group of workers with no queen and no brood. Nevertheless, it is important to note that even with our minimalistic conditions, we are able to capture such features as spatial segregation between caste activity even if this effect can be more pronounced at larger scale^46^. Finally, theoretical investigations could shed light on the still unclear relationship between the spatial behaviour of individuals and the subsequent food dissemination dynamics^47^.

## Supplementary information

Related Manuscript File

Dataset 1

## Data Availability

All data generated or analysed during this study are included in this published article (and its Supplementary Information files).
