# Supplementary material for: A spatiotemporal analysis of the food dissemination process and the trophallactic network in the ant Lasius niger
Source: Sci Rep. 2019 Oct 30;9:15620. doi: 10.1038/s41598-019-52019-6 (PMC6821819; doi:10.1038/s41598-019-52019-6)
Supplement: Supplementary file 1 — Related Manuscript File [file 41598_2019_52019_MOESM1_ESM.pdf]

Supplementary Information

A spatiotemporal analysis of the food dissemination process and the trophallactic network in the ant *Lasius niger*

Joffrey Planckaert[1], Stamatios C. Nicolis[1], Jean-Louis Deneubourg[1], Cédric Sueur[2] and Olivier Bles[1*]

[1]Center for Nonlinear Phenomena and Complex Systems (Cenoli) - CP 231, Université libre de Bruxelles (ULB), Campus Plaine, Boulevard du Triomphe, Building NO - level 5, B-1050 Bruxelles, Belgium

[2]Université de Strasbourg, CNRS, IPHC, UMR 7178, Strasbourg, France

*olivier.bles@ulb.ac.be

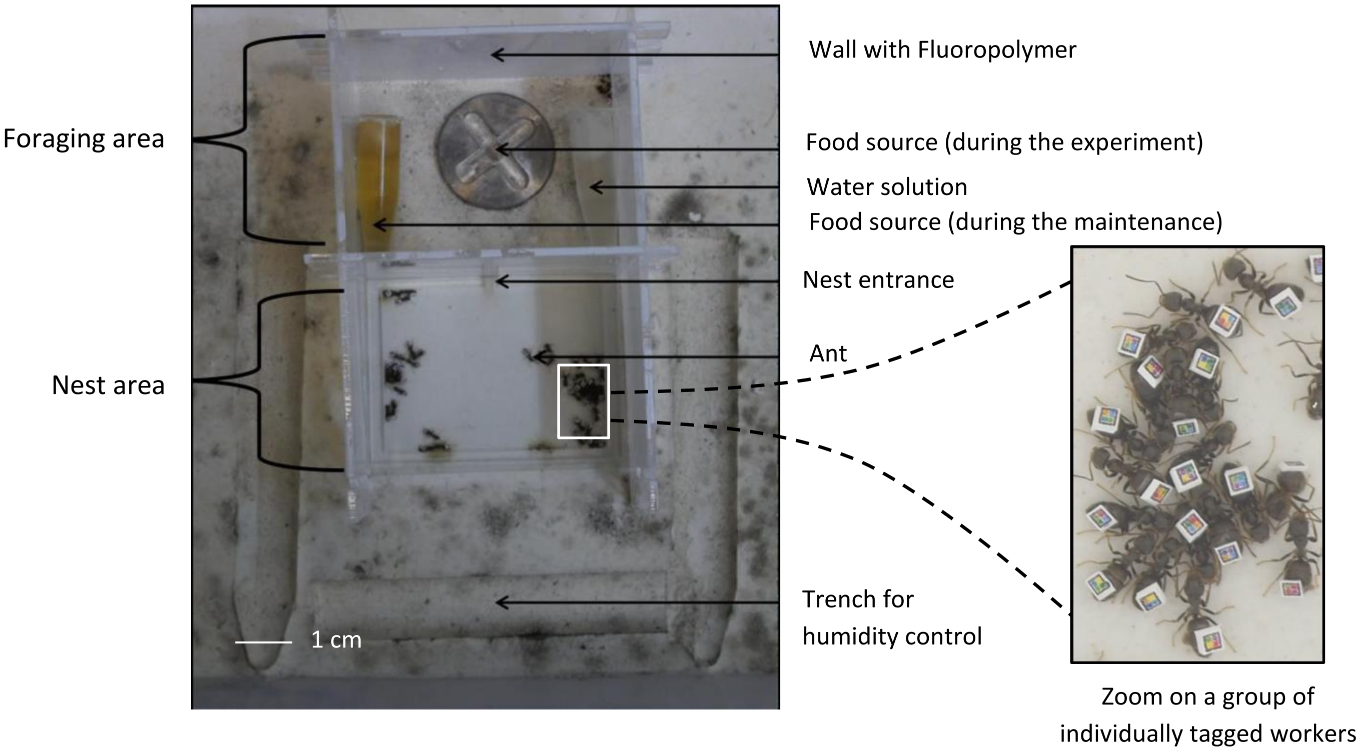

Figure S1. Top view of the experimental setup.

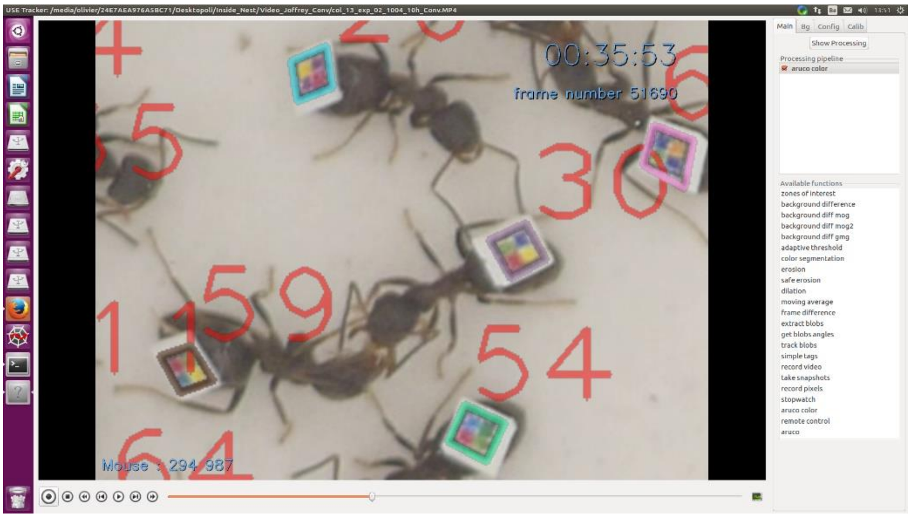

Figure S2. Screen capture for the tracking software USETracker illustrating the individual 50 receiving food from the individual 30. The individual 30 opened his mandibules and displays a droplet of sucrose solution between them while the individual 59 turns and moves forward his head to receive it.

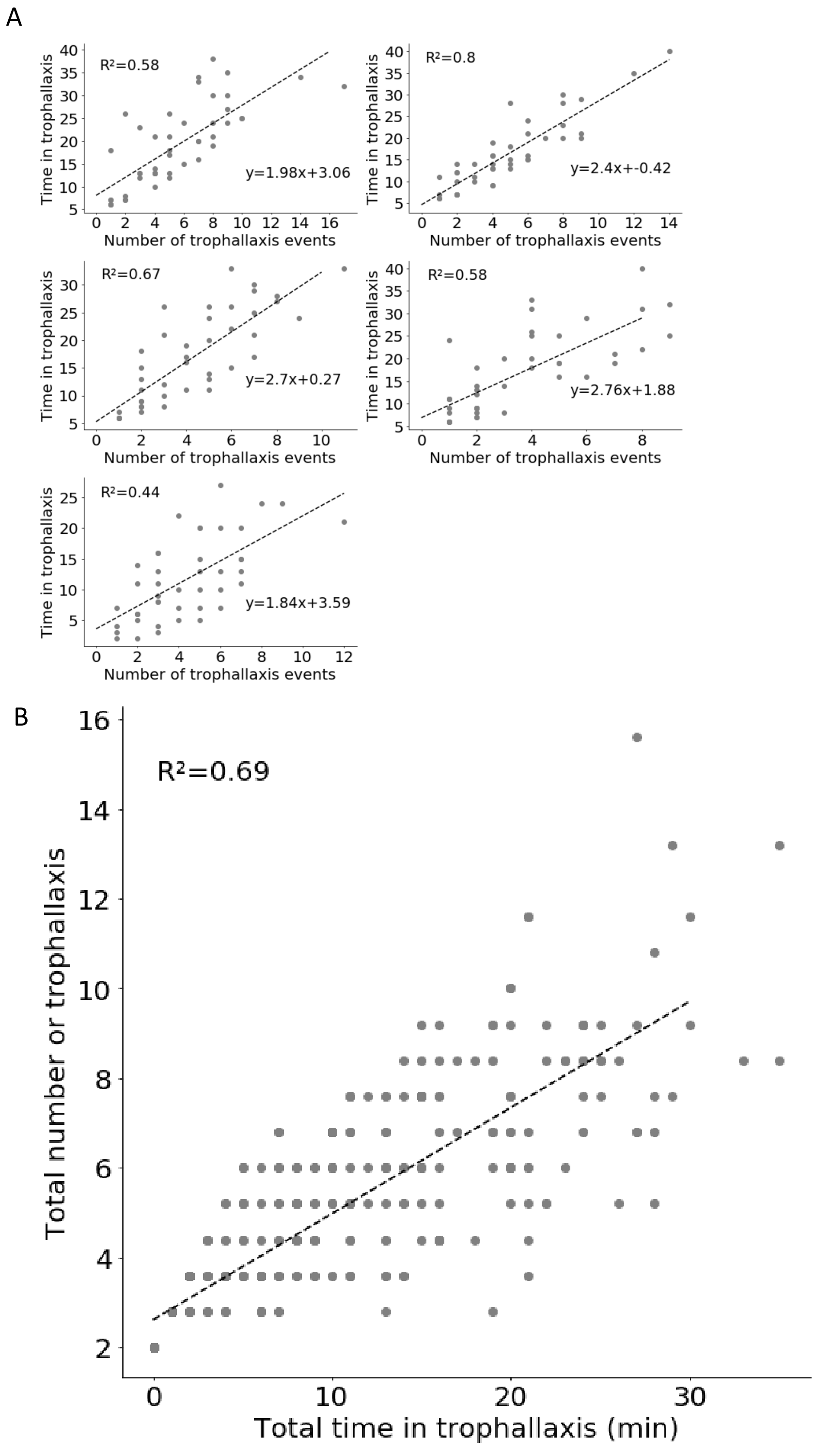

Figure S3. Correlation between the number of trophallactic events and the time spent in trophallactic interactions. **A.** For each subcolony **B.** Mean from the five subcolonies merged.

Subcolonies	1	2	3	4	5
Total number of ants	58	45	50	57	57
Number of active ants	47	43	43	44	44
Number of foragers	14	11	14	9	13
Number of non foragers	44	34	36	48	44
Number of initiator	35	34	28	27	34
Number of recipients	41	40	36	35	40
Total number of trophallactic events	129	104	90	77	95
Trophallactic event given by foragers	89	52	62	47	51
Mean trophallactic event given by F	6.36	4.73	4.77	5.22	4.25
Standard deviation of trophallactic events given by foragers	3.22	2	2.26	2.62	1.69
Trophallactic event given by F	40	52	28	30	44
Mean trophallactic event given by NF	1.21	1.62	0.93	0.86	1.38
Standard deviation of trophallactic events given by foragers	1.49	2.01	1.18	0.96	1.41
Statistic Mann-whitney trophallactic events given between foragers and non foragers	26	43	39.5	32.5	31.5
p-value Mann-whitney trophallactic events given between foragers and non foragers	0	0.00008	0.00001	0.00008	0.00001
Trophallactic event received by foragers	32	16	13	8	17
Mean trophallactic received given by F	2.29	1.45	1	0.89	1.42
Standard deviation of trophallactic events received by foragers	2.02	1.08	1.11	1.1	1.19
Trophallactic event received by NF	97	88	77	69	78
Mean trophallactic received gave by NF	2.94	2.75	2.57	1.97	2.44
Standard deviation of trophallactic received given by foragers	1.61	1.94	1.8	1.46	1.32
Statistic Mann-whitney trophallactic events received between foragers and non foragers	190.5	97	82.5	88.5	109
p-value Mann-whitney trophallactic events received between foragers and non foragers	0.17245	0.01222	0.00124	0.02032	0.01271
Mean of the degree for foragers	0.19	0.15	0.14	0.14	0.13
Standard deviation of foragers degree	0.07	0.05	0.05	0.06	0.04
Mean of non foragers degree	0.09	0.1	0.08	0.07	0.09
Standard deviation of the degree for non foragers	0.05	0.07	0.05	0.04	0.06
Statistic of Mann Whitney for the degree between foragers and non foragers	62.5	94	89.5	49.5	90
p-value of Mann Whitney for the degree between foragers and non foragers	0.00004	0.01104	0.00253	0.00073	0.00344
Mean of the betweenness for foragers	0.06	0.06	0.06	0.1	0.06
Standard deviation of foragers betweenness	0.04	0.05	0.03	0.08	0.04
Mean of non foragers betweenness	0.02	0.04	0.03	0.03	0.03
Standard deviation of the betweenness for non foragers	0.03	0.05	0.04	0.05	0.05

**Statistic of Mann Whitney for the betweenness between foragers and non foragers**	95	118	88.5	70.5	101
***p-value* of Mann Whitney for the betweenness between foragers and non foragers**	0.0008	0.05437	0.0025	0.00495	0.00847
**Mean of the closeness for foragers**	0.35	0.28	0.35	0.46	0.34
**Standard deviation of foragers closeness**	0.11	0.05	0.16	0.24	0.07
**Mean of non foragers closeness**	0.29	0.21	0.33	0.38	0.28
**Standard deviation of the closeness for non foragers**	0.34	0.23	0.38	0.4	0.31
**Statistic of Mann Whitney for the closeness between foragers and non foragers**	142.5	78	147	132.5	95
***p-value* of Mann Whitney for the closeness between foragers and non foragers**	0.01944	0.0032	0.09947	0.23063	0.00525
**Mean of the eigenvector for foragers**	0.18	0.15	0.18	0.2	0.18
**Standard deviation of foragers eigenvector**	0.08	0.08	0.06	0.1	0.06
**Mean of non foragers eigenvector**	0.1	0.12	0.11	0.1	0.11
**Standard deviation of the eigenvector for non foragers**	0.07	0.09	0.07	0.07	0.07
**Statistic of Mann Whitney for the eigenvector between foragers and non foragers**	104	128	92	65	73
***p-value* of Mann Whitney for the eigenvector between foragers and non foragers**	0.00163	0.09305	0.00336	0.00371	0.0009

Table S1. Overview of the different characteristics of the five colonies.

	Kruskall-Wallis	
Indices	*H*	*p*
Degree	0.70	0.95
Out Degree	1.80	0.77
In Degree	0.66	0.95
Betweenness	9.10	0.06
Closeness	4.0	0.41
Eigenvector	0.93	0.91
	Chi-square	
	χ^2	*p*
Ratio F/NF	2.4	0.64

Table S2. Homogeneity of trophallactic networks characteristics and castes.

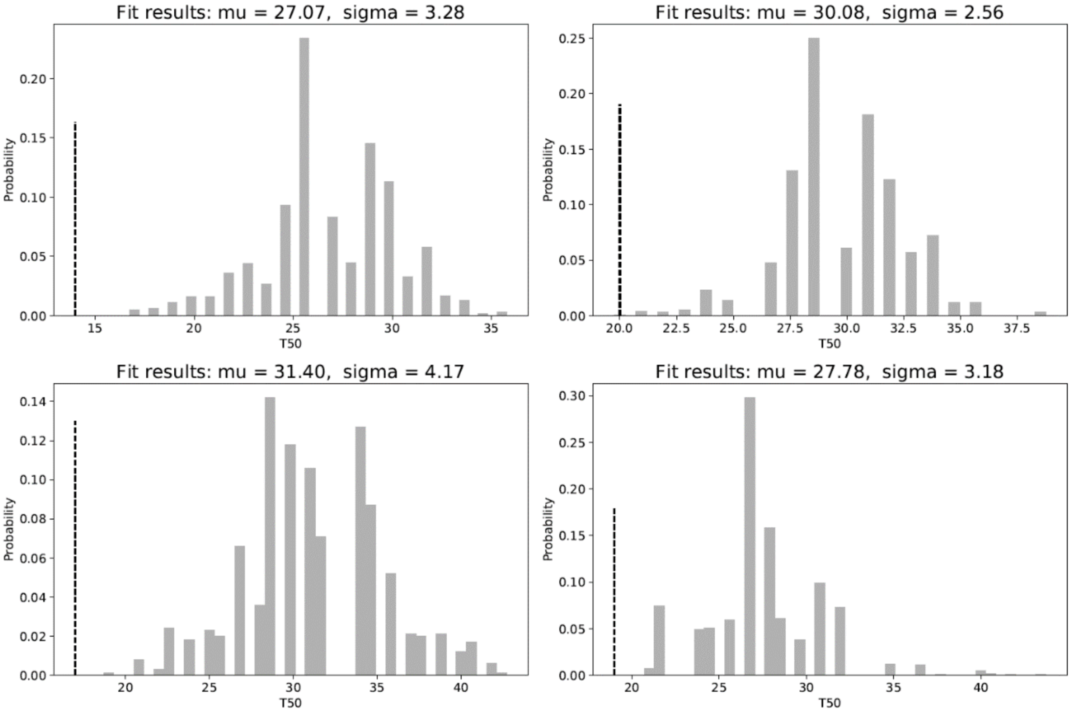

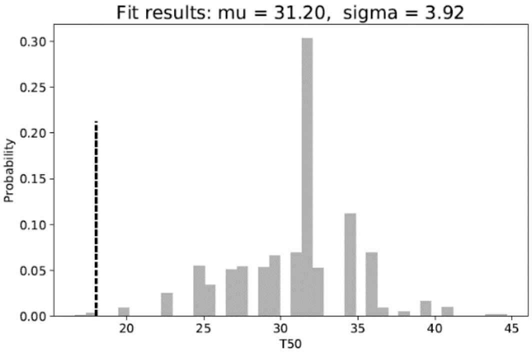

	Z-value	p-val
Exp 1	-4.51	<0.0001
Exp 2	-4.72	<0.0001
Exp 3	-3.91	<0.0001
Exp 4	-3.56	0.0003
Exp 5	-4.13	<0.0001

Figure S4. T_{50} needed to half the population performed at least one trophallactic event in each experiment (vertical dashed line) and in the corresponding randomized network (grey bar, N=1000). Table shows the statistical values of the Z-Test comparing each experimental value to the distributions of the randomized ones for in each colony.

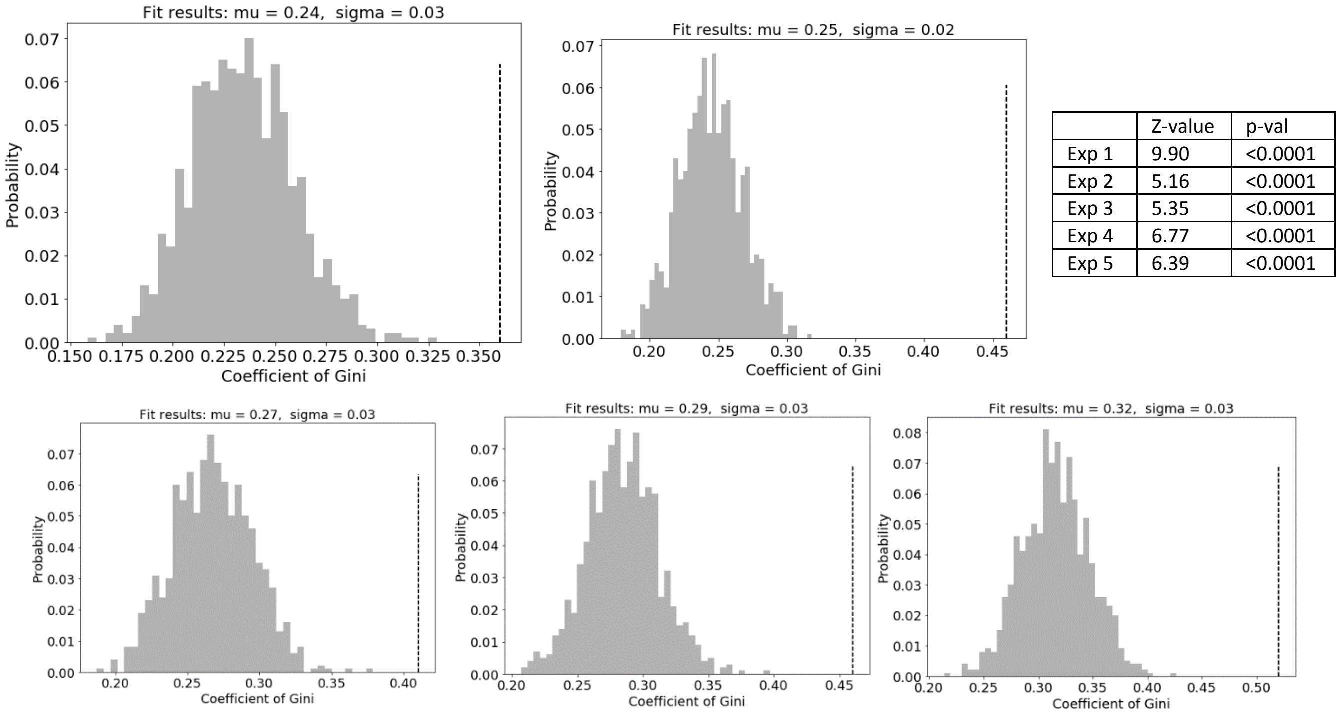

	Z-value	p-val
Exp 1	9.90	<0.0001
Exp 2	5.16	<0.0001
Exp 3	5.35	<0.0001
Exp 4	6.77	<0.0001
Exp 5	6.39	<0.0001

Figure S5. Gini coefficient experimentally measured value of all active ants (vertical dotted line) and the distribution from the FR randomized networks (grey bars, N=1000) for each colony. Table shows the statistical values of the Z-Test comparing each experimental value to the distribution of the randomized one for in each colony.

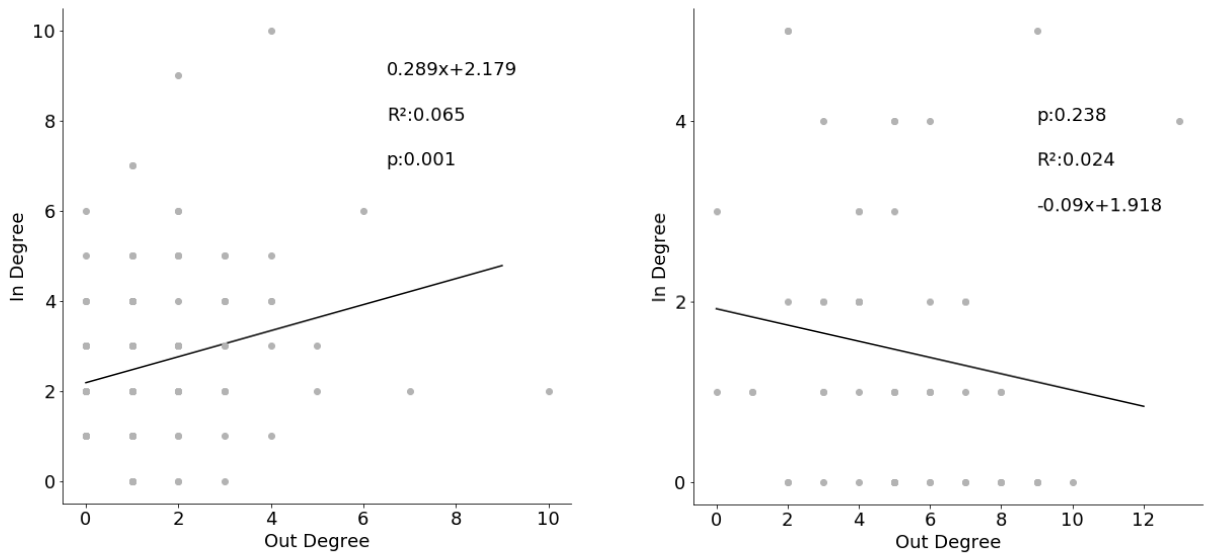

Figure S6. Correlation between the number of given and received trophallactic interactions per individual. **A.** Non-foragers. **B.** Foragers.

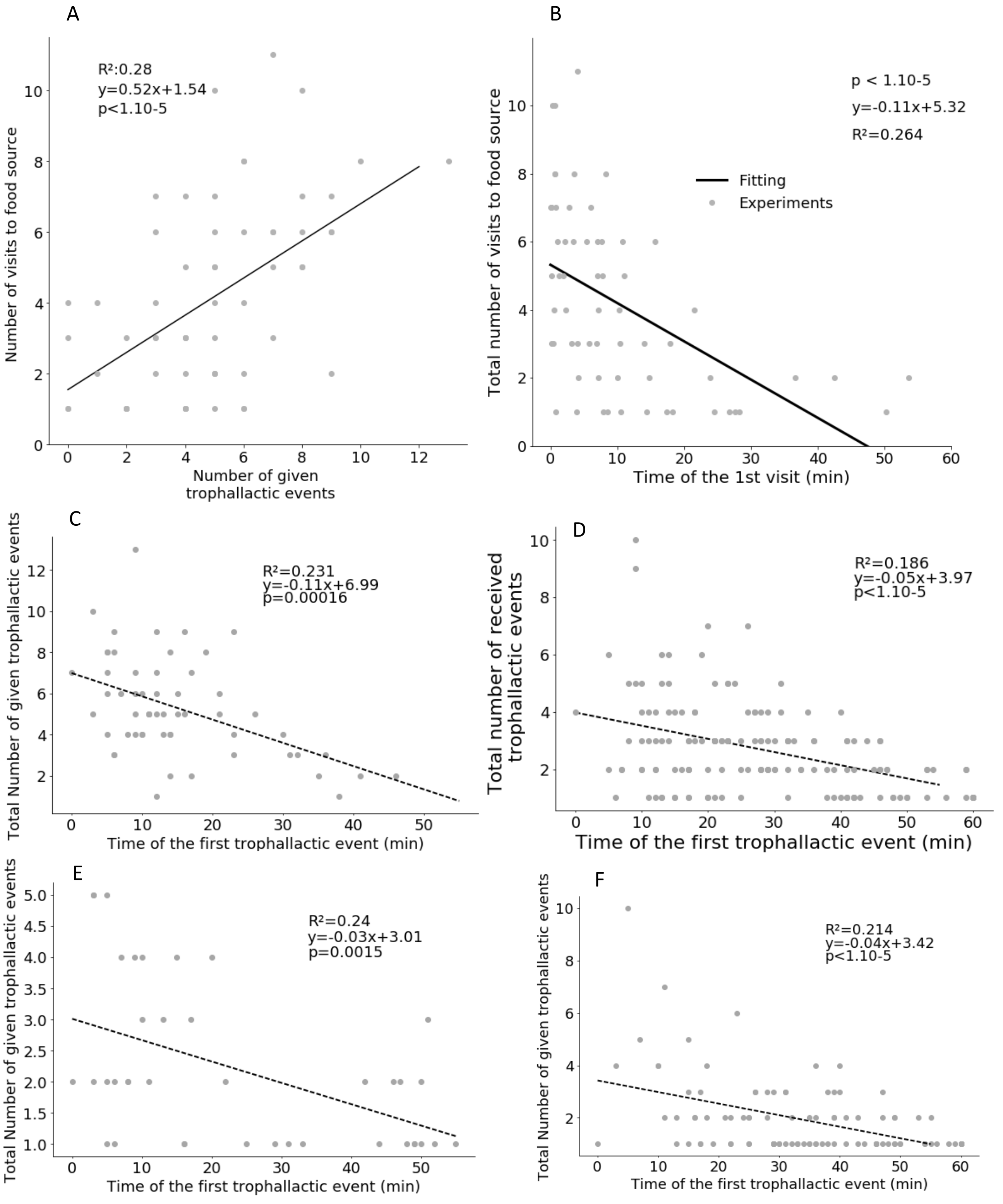

Figure S7.A. Correlation between the number of visits to the food source and the number of given trophallactic events. **B.** Correlation between the time of the 1st visit to food source and the total number of visits to food source. **C.** Correlation between the total number of given trophallactic events per forager and the time it performed its first trophallactic exchange as donor. **D.** Correlation between the total number of received trophallactic events per non forager and the time it performed its first trophallactic exchange as receiver. **E.** Correlation between the total number of received trophallactic events per forager and the time it performed its first trophallactic exchange as receiver. **F.** Correlation between the total number of given trophallactic events per non forager and the time it performed its first trophallactic exchange as donor.

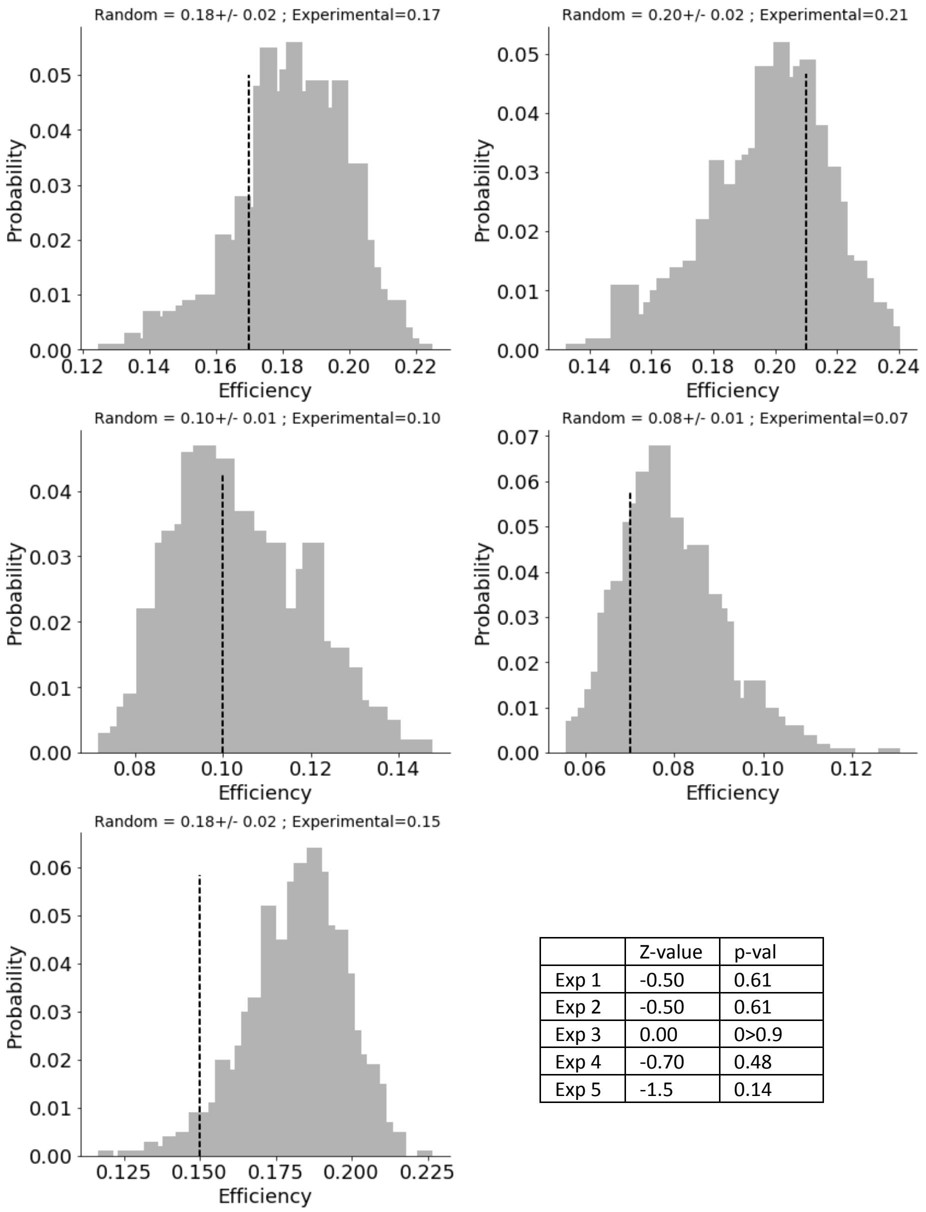

	Z-value	p-val
Exp 1	-0.50	0.61
Exp 2	-0.50	0.61
Exp 3	0.00	0>0.9
Exp 4	-0.70	0.48
Exp 5	-1.5	0.14

Figure S8. Efficiency coefficient experimentally measured (vertical dotted line) and the distribution from FR randomized networks (grey bars, N=1000) for each colony. Table shows the statistical values of the Z-Test comparing each experimental value to the distribution of the randomized one for in each colony.

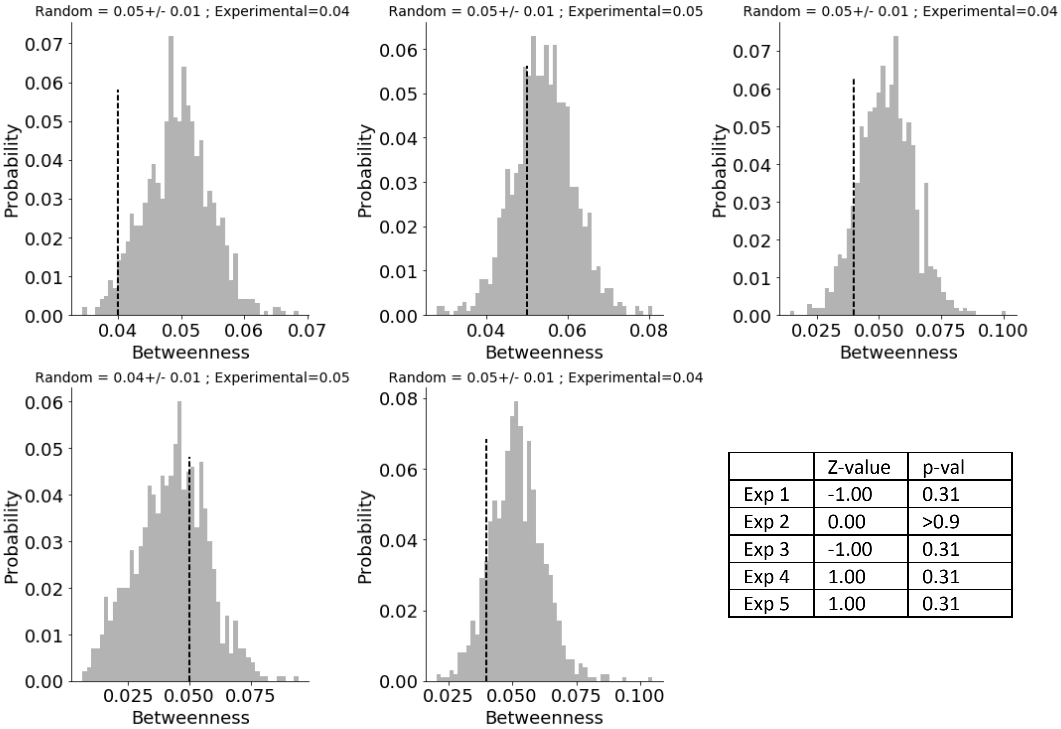

	Z-value	p-val
Exp 1	-1.00	0.31
Exp 2	0.00	>0.9
Exp 3	-1.00	0.31
Exp 4	1.00	0.31
Exp 5	1.00	0.31

Figure S9. Betweenness coefficient experimentally measured (vertical dotted line) and the distribution from FR randomized networks (grey bars, N=1000) for each colony. Table shows the statistical values of the Z-Test comparing each experimental value to the distribution of the randomized one for in each colony.

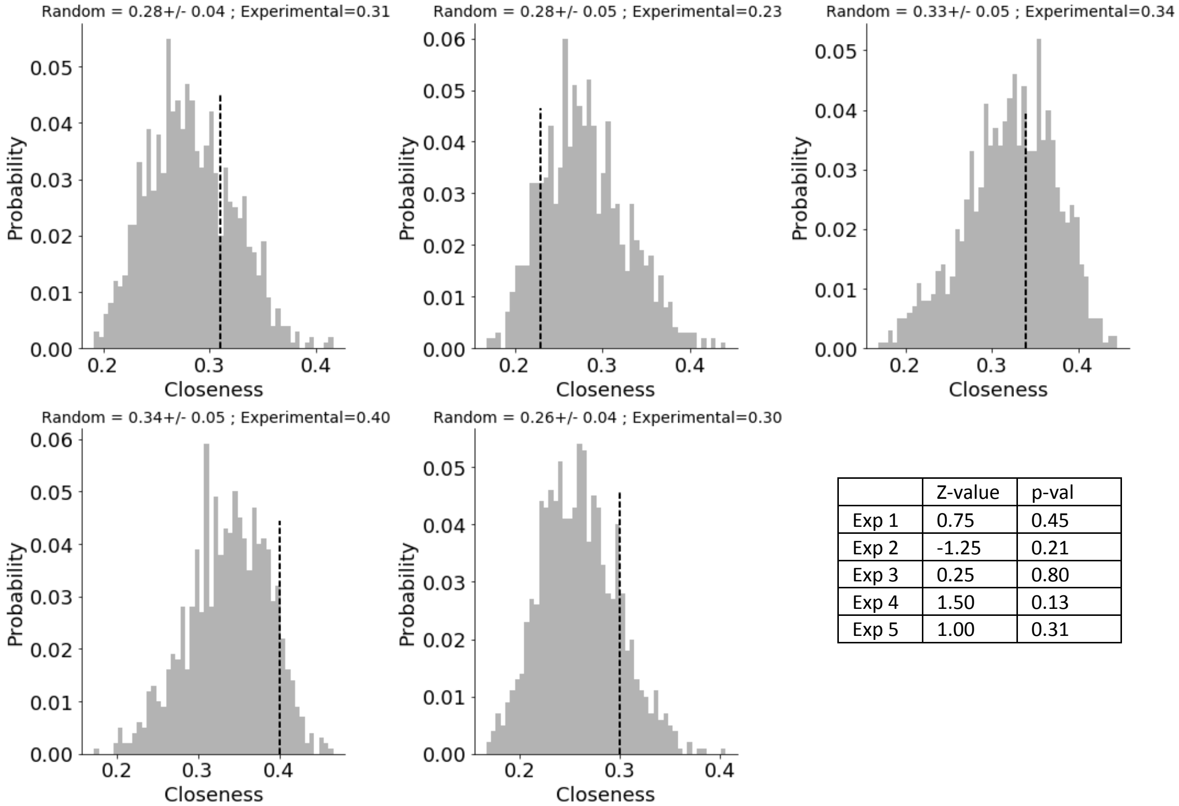

	Z-value	p-val
Exp 1	0.75	0.45
Exp 2	-1.25	0.21
Exp 3	0.25	0.80
Exp 4	1.50	0.13
Exp 5	1.00	0.31

Figure S10. Closeness coefficient experimentally measured (vertical dotted line) and the distribution from FR randomized networks (grey bars, N=1000) for

each colony. Table shows the statistical values of the Z-Test comparing each experimental value to the distribution of the randomized one for in each colony.

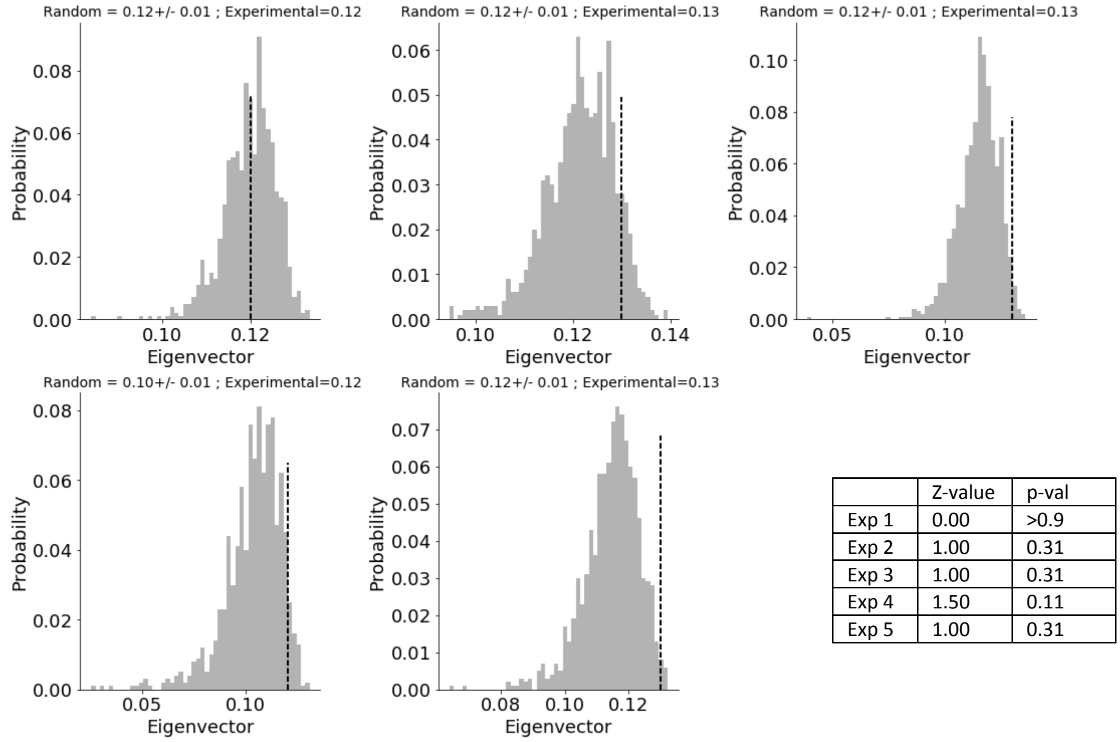

	Z-value	p-val
Exp 1	0.00	>0.9
Exp 2	1.00	0.31
Exp 3	1.00	0.31
Exp 4	1.50	0.11
Exp 5	1.00	0.31

Figure S11. Eigenvector coefficient experimentally measured (vertical dotted line) and the distribution from FR randomized networks (grey bars, N=1000) for each colony. Table shows the statistical values of the Z-Test comparing each experimental value to the distribution of the randomized one for in each colony.

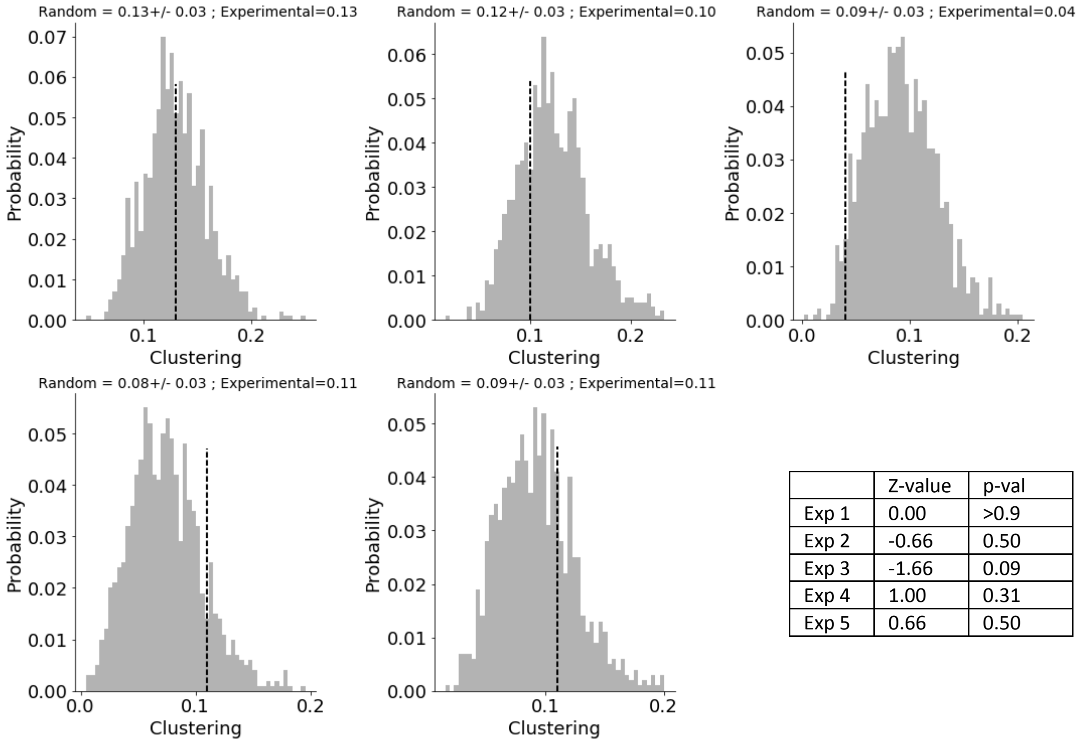

	Z-value	p-val
Exp 1	0.00	>0.9
Exp 2	-0.66	0.50
Exp 3	-1.66	0.09
Exp 4	1.00	0.31
Exp 5	0.66	0.50

Figure S12. Clustering coefficient experimentally measured (vertical dotted line) and the distribution from FR randomized networks (grey bars, N=1000) for each colony. Table shows the statistical values of the Z-Test comparing each experimental value to the distribution of the randomized one for in each colony.

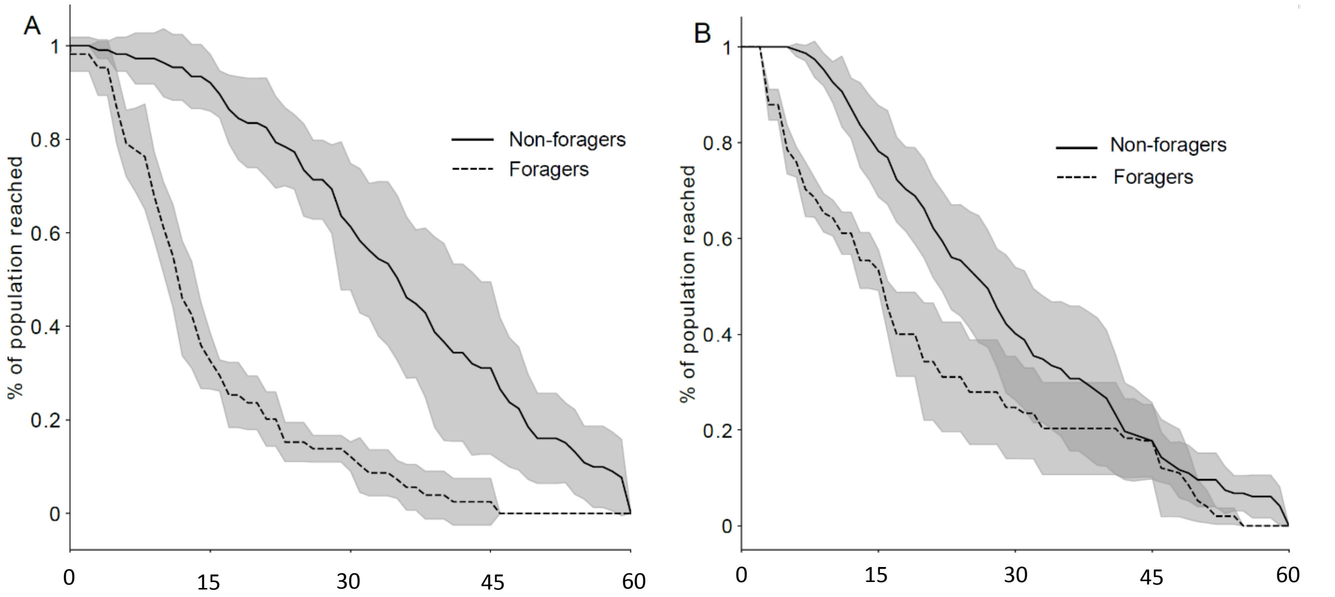

Figure S13.A. Survival curve of the first given trophallactic event (foragers=dotted line; non-foragers=full line) **B.** Survival curve of the first received trophallactic event (foragers=dotted line; non-foragers=full line).

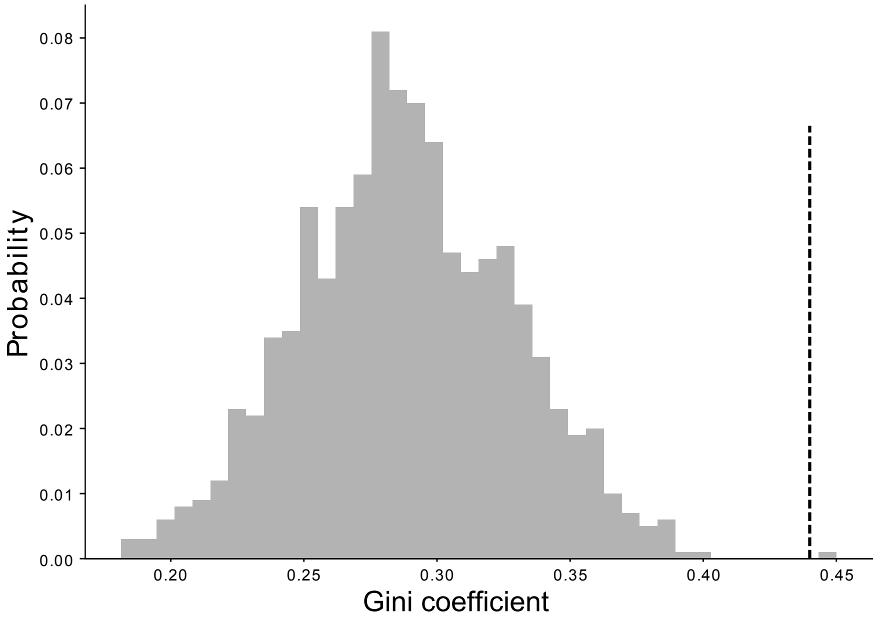

Figure S14. Gini coefficient from the mean number of trophallactic events in each of the 30 cells (see material and methods in the main manuscript) from experimental nests (vertical dotted bar) and from a random spatial distribution of the trophallactic events (grey bar, N=1000, ZT, $p < 0.0002$).

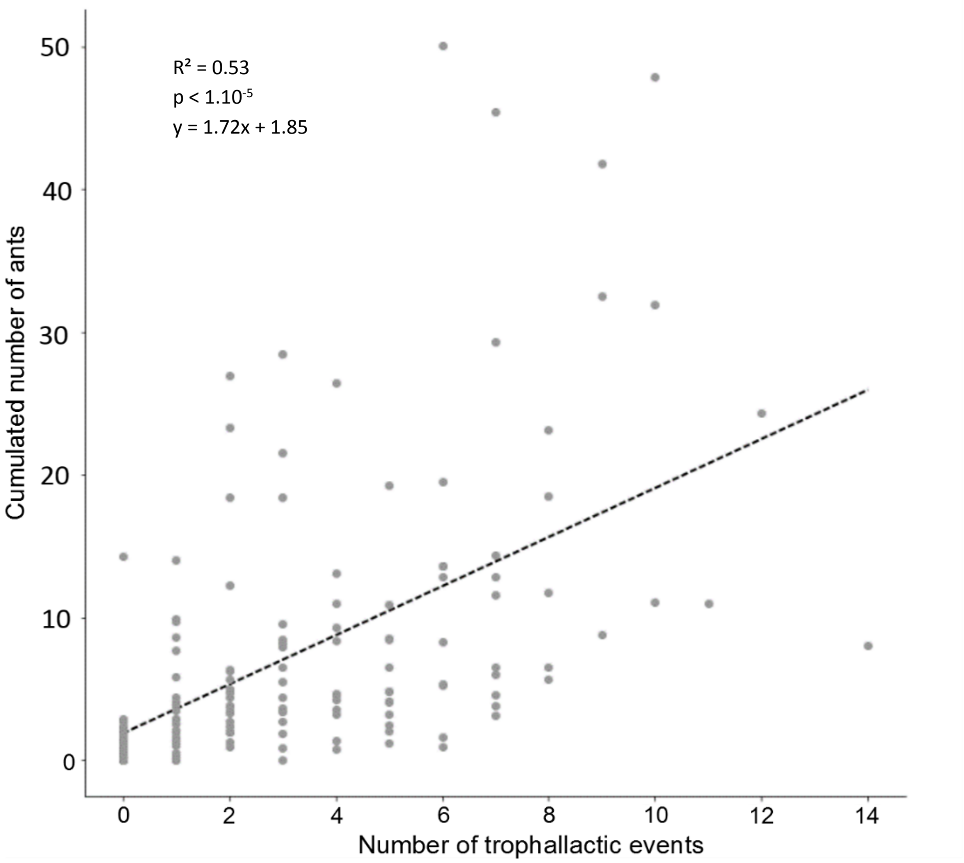

Figure S15. Correlation between the number of trophallactic events per cell and the cumulative number of ants in the corresponding cell (see Materials and Methods section for details).
